# A combination sampling approach for epidemiologic research in humanitarian settings: a case analysis of a study of depressive disorder prevalence among refugees in Greece

**DOI:** 10.1186/s12889-021-10342-6

**Published:** 2021-02-02

**Authors:** Danielle N. Poole, Nathaniel A. Raymond, Jos Berens, Mark Latonero, Julie Ricard, Bethany Hedt-Gauthier

**Affiliations:** 1 Neukom Institute for Computational Science, Dartmouth College, Hanover, NH 03755 USA; 2 Department of Global Health and Population, Harvard T.H. Chan School of Public Health, 665 Huntington Avenue, Boston, MA 02115 USA; 3 Harvard Humanitarian Initiative, Harvard T.H. Chan School of Public Health, 14 Story Street, Cambridge, MA 02138 USA; 4 Jackson Institute of Global Affairs, Yale University, 55 Hillhouse Avenue, New Haven, CT 06520 USA; 5 Centre for Innovation, Leiden University, Den Haag, 2511 VA The Netherlands; 6 Data & Society Research Institute, New York, NY 10011 USA; 7 Data-Pop Alliance, New York, NY 10016 USA; 8 Department of Global Health and Social Medicine, Harvard Medical School, 641 Huntington Avenue, Boston, MA 02115 USA; 9 Department of Biostatistics, Harvard T.H. Chan School of Public Health, 665 Huntington Avenue, Boston, MA 02115 USA

**Keywords:** Humanitarian research, Research ethics, Refugees, Global mental health

## Abstract

**Background:**

Understanding the burden of common mental health disorders, such as depressive disorder, is the first step in strengthening prevention and treatment in humanitarian emergencies. However, simple random sampling methods may lead to a high risk of coercion in settings characterized by a lack of distinction between researchers and aid organizations, mistrust, privacy concerns, and the overarching power differential between researchers and populations affected by crises. This case analysis describes a sampling approach developed for a survey study of depressive disorder in a Syrian refugee camp in Greece (*n* = 135).

**Discussion:**

Syrian refugees face an extraordinarily high burden of depressive disorder during the asylum process (43%), necessitating population screening, prevention, and treatment. In order to preserve the informed consent process in this refugee camp setting, the research team developed a two-phase sampling strategy using a map depicting the geographical layout of the housing units within the camp. In the first phase, camp management announced a research study was being undertaken and individuals were invited to volunteer to participate. The participants’ container (housing) numbers were recorded on the map, but were not linked to the survey data. Then, in the second phase, the camp map was used for complementary sampling to reach a sample sufficient for statistical analysis. As a result of the two phases of the sampling exercise, all eligible adults from half the containers in each block were recruited, producing a systematic, age- and sex-representative sample.

**Conclusions:**

Combining sampling procedures in humanitarian emergencies can reduce the risk of coerced consent and bias by allowing participants to approach researchers in the first phase, with a second phase of sampling conducted to recruit a systematic sample. This case analysis illuminates the feasibility of a two-phase sampling approach for drawing a quasi-random, representative sample in a refugee camp setting.

## Background

### Humanitarian context

In 2015, an unprecedented 1 million asylum seekers crossed the Mediterranean Sea to Europe, including more than 450,000 Syrian refugees [1]. At the same time, the duration of asylum-seeking procedures in Greece increased - with application processing lasting an average of over 1 year [2].

Despite the increased time spent in the asylum process, there is a lack of evidence documenting the health needs of individuals in the transit phase of forced migration. Existing research suggests that the health needs of forced migrants may evolve over time in response to policy changes and border closures [3], as well as throughout the course of the forced migration journey [4]. In particular, the mental health needs of populations displaced by humanitarian emergencies remain a significant yet often overlooked public health problem [5], and are exacerbated by pre- and post-migration stressors [6].

### Research study

This case analysis describes an observational study of mental health among asylum seekers in a Syrian refugee camp in the Attica region of Greece. The research question, study design, and interpretation of the results were informed by a multisectoral partnership, including academics (Harvard University, Leiden University), an intergovernmental organization (International Organization for Migration), non-governmental organizations (International Data Responsibility Group, Data & Society Research Institute, and the Data-Pop Alliance), and local municipality governments. During the study period of January–February 2017, camp inhabitants lived in shipping containers (“containers”) with running water and electricity, meals provided by the Greek Airforce, and public toilet and shower facilities.

### Study design

This study included a cross-sectional, face-to-face survey designed to estimate the prevalence of major depressive disorder (MDD), its risk factors, and associations with mobile phone connectivity. Major depressive disorder was classified by the Patient Health Questionnaire-8 (PHQ-8), an eight-item screening measure used extensively to screen for MDD in epidemiologic research [7, 8] when the assessment of suicidal ideation is not a primary outcome and may cause distress [7, 9]. The PHQ-8 has been validated in myriad settings and languages, including among migrant populations [10, 11] and in Arabic [9, 12]. Moreover, the detection of MDD by the persistence and severity of depressive symptoms for 2 weeks [13] is an important threshold for clinical diagnostic assessments and treatment [14]. In our study, the PHQ-8 had a Cronbach’s α of 0.77, indicating satisfactory reliability of this scale within the study population.

The survey instrument also assessed sociodemographic and displacement characteristics, mobile phone connectivity, and perceptions of privacy. The survey instrument was translated and back-translated into Arabic. Arabic- and English-speaking translators paired with members of the research team administered the survey to participants via digital tablets.

The study population included all Arabic- and/or English-speaking adults residing in the camp at the time of the study. A minimum sample size of 97 was calculated based on the specific aim of estimating the prevalence of MDD with maximum variability in the binary outcome and ± 10% precision in the 95% Confidence Intervals (95% CIs), which was considered sufficient given the wide range of MDD prevalence previously reported in refugee populations [15, 16].

This study received ethics review by the International Organization for Migration (IOM) Greek research ethics advisory board and the Institutional Review Board of the Harvard T.H. Chan School of Public Health (IRB-16-2015). Oral informed consent was obtained due to the potential risk to participants if their identities were uncovered via consent forms containing their signatures, consistent with the criteria for oral informed consent outlined in United States federal regulation 45 CFR 46 [17].

## Results

A total of 135 individuals participated in the study, representing 40% of the adult population. The median age of participants was 30 years (Interquartile range [IQR]: 24–37 years), 41% of the sample were women, 74% had married, 67% had children, and 77% had attended some secondary school. The median time displaced was 12 months (IQR: 11–36 months) and participants had spent a median of 10 months in Greece (IQR: 10–11 months).

An extraordinarily high burden of depressive symptoms was identified – the prevalence of MDD classified by the PHQ-8 was 44% [18]. Risk factors for MDD classification included being a woman (Adjusted Odds Ratio [AOR]: 3.32, 95% CI: 1.12–8.62), each additional child (AOR: 1.61, 95% CI: 1.15–2.25), and each additional month in the asylum process (AOR: 1.15, 95% CI: 1.00–1.31). Ever being married was a protective factor for the probability of MDD classification (AOR: 0.23, 95% CI: 0.05–0.95).

## Discussion

### Scientific importance

Depressive disorder is among the most common mental disorders, affecting 5% of the global population, and a leading cause of disability worldwide [19]. The development of depression in transit is likely to undermine individual and societal functioning, which are essential for survival and resettlement [20]. Depression is also likely to lead to adverse acculturation outcomes [21]. Finally, the effects of depression compound intergenerationally – parental depression has been associated with negative and withdrawn parenting and poor child physical and psychosocial health [22], while remission improved functioning in children in the general population [23].

And yet, there is a lack of evidence describing the burden and risk factors of depression in humanitarian settings, particularly during the transit phase of forced migration. While previous studies found significantly higher rates of post-traumatic stress disorder among Syrian migrants compared to host populations [24, 25], emphasis on pre-migration trauma may overshadow the current psychological needs of asylum seekers and the roles of displacement-related stressors [26, 27], which have been shown to contribute to the development of chronic mental health disorders, including depressive disorders [6, 28, 29]. Furthermore, emphasis on past trauma may result in failure to consider the effects of current life circumstances on mental health [30, 31].

This study provided the first evidence of the prevalence and risk factors of MDD among Syrian refugees seeking asylum in Greece. All participants reporting symptoms consistent with MDD were referred to an onsite psychologist. The high prevalence of MDD demonstrated the importance of the psychosocial services available in the camp, and reveals the unique health challenges of the transit phase of forced migration.

The finding of an unacceptably high burden of MDD supports the theory that the “healthy migrant effect” – the phenomenon that migrants are on average healthier than their host populations – does not apply to migrants displaced by conflict or natural disaster [32]. In contrast, forced migrants face higher rates of health problems, including mental health disorders [33]. The incorporation of screening and treatment into service provision for forcibly displaced populations is urgently needed to mitigate the effects of time in camps as governments work to address protracted asylum procedures.

Evidence of the burden of depressive symptoms during the transit phase of migration in the context of protracted asylum procedures was disseminated to operational and academic stakeholders alike. The results were communicated to IOM camp management and headquarters, in a publicly available report [34], conference proceedings [35, 36], and in peer-reviewed journal articles [18, 37]. The translation of the study results into practice included a performance evaluation of the previously validated mental health instruments in a sequential screening approach for the classification of MDD, which preserved classification accuracy while reducing the participant response burden [37].

### Research challenges

Beyond evidence of the burden and risk factors of depressive disorder, this study generated a novel methodological sampling approach, motivated by the ethical challenges of conducting human subjects research in humanitarian emergencies. As a pillar of ethical research, the right to provide informed consent to participate in human subjects research is derived from three mutually reinforcing sources: the Nuremberg Code [38], the Declaration of Helsinki [39], and the Belmont Report [40]. In humanitarian contexts, the principle of humanity encoded in the humanitarian charter further extends the right of populations in humanitarian emergencies to informed consent [41–43]. However, the obligation of informed consent is often unheeded: in a systematic review of studies undertaken in Darfur (*n* = 68), only 43% reported obtaining informed consent. Moreover, empirically valid, alternative recruitment and sampling procedures responsive to the ethical challenges of humanitarian emergencies are lacking [44].

While it is incumbent on researchers to design study procedures that preserve the principles of ethical research [45] while limiting bias with representative sampling, the accuracy and internal generalizability of research findings in humanitarian settings are often impeded by nonrepresentative samples or ambiguous sampling strategies [46]. This lack of evidence demonstrating the consequences of participant recruitment and sampling strategies on internal generalizability in complex settings with similar challenges remains a methodological lacuna, in part due to the lack of detail in reporting procedures [46].

In this study, the recruitment and sampling strategies were adapted to preserve ethical and empirical rigor in response to the following ethical challenges: 1) lack of distinction between the researchers and aid organizations collecting data, 2) potential mistrust of researchers, 3) protection of participant privacy, and 4) overarching power differential between the researchers and study participants who were experiencing a crisis.

### Lack of distinction

Research in humanitarian emergencies is often undertaken by multisectoral study teams, including representatives from humanitarian organizations. Moreover, humanitarian organizations increasingly collect quantitative data [47], rendering human subjects research activities indistinguishable from humanitarian monitoring and evaluation and needs assessments. Potential conflation of study procedures and the activities of humanitarian responders may lead to the perception of participation in research having bearing on access to aid.

### Mistrust of researchers

Mistrust of health research is a significant barrier to study participation [48], and may be exacerbated in emergency settings [49]. In this study, camp residents were unfamiliar with the research team, the purpose of the study, and their right to decline participation, representing possible barriers to trust.

### Participant privacy

In order to protect the privacy of the participants, the research team chose not to collect personally identifiable information (PII) (i.e. name, date of birth) due to the potential risk to participants if their identities and residence in the camp were discovered. The sensitive nature of PII in this population was confirmed in the study findings – 40% of participants considered their name to be sensitive or very sensitive information, while 22% considered their date of birth to be sensitive or very sensitive information [34]. The decision to minimize data collection was also responsive to the humanitarian context, in which nearly one-third (31%) of participants reported being asked to provide information about themselves that they would rather not since leaving their home country. Additionally, despite perceptions that digital data collection is less personal [50], data collection via digital platforms includes potentially identifying meta-data and is subject to security breaches [51].

### Power asymmetries

As a dynamic and unstable relationship intersecting with knowledge [52], the power dynamics between researchers and populations dependent on humanitarian aid may result in excessive pressure to participate. It is therefore incumbent on researchers to recognize the power hierarchies emergent in engagement with crisis-affected populations [47].

Together, the potential lack of distinction between the researchers and aid organizations collecting data, mistrust, privacy concerns, and the overarching power differential between researchers and the study population experiencing a crisis were expected to have a significant impact on the informed consent process, and potentially the representativeness of the study sample.

The researcher team decided that the risk that simple random sampling (SRS) would exacerbate these challenges, thereby compromising the informed consent process and representativeness of the sample, was unethical and empirically unacceptable. Thus, despite the availability of a list of camp residents, a novel methodological approach was developed and validated for this study setting without reliance on a detailed sampling frame and the collection of PII, in order to preserve the informed consent process while drawing a representative sampling.

### Research strategies

#### Sampling method innovation

In consultation with IOM camp management, the research team developed a two-phase sampling approach with the overall goal of obtaining a quasi-random, representative sample without reliance on SRS. The first phase consisted of purposive sampling in order to protect the voluntary nature of participation and was designed to establish trust within the camp community [53]. Camp management announced that a research study was being undertaken on the topic of migrant health and adults were invited to volunteer. While no PII was recorded, a camp map depicting the containers (housing units) organized into blocks was used to facilitate alternative sampling in a future phase. The participants’ container numbers were marked on a camp map, but were not linked to the survey data. A total of 90 individuals were recruited in the first phase of sampling.

A second phase of sampling was deemed feasible upon receiving positive feedback from camp management and inhabitants. The camp map was used to conduct systematic geographical sampling. The occurrence of depression was expected to be randomly distributed throughout the camp. Thus, a geographically distributed sample was expected be both representative of the camp population and the prevalence of MDD.

**Fig. 1:**
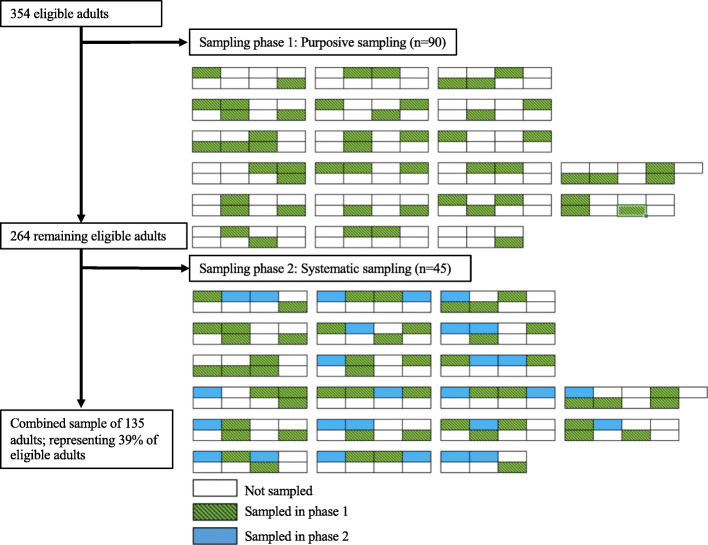
Two-phase sampling approach

A systematic sample was drawn using the camp map to recruit all eligible adults from exactly half the containers in each block. Based on the container locations of participants in the first phase of sampling, the research team identified blocks that were underrepresented. Starting at the north-west container of the underrepresented blocks, the research team recruited adults to participate in the study. Neighbouring containers were sampled, moving from west to east, until individuals from exactly half the containers in each block were recruited to participate in the study, producing an even distribution of sampled containers across the camp. Forty-five participants were included in the second phase of sampling, with a response rate of 92%. An illustration of the mixed sampling strategy is presented in Fig. 1. The total combined sample from the first and second phases of sampling was 135 participants.

#### Validation of the two-phase sampling approach

Validation is a key challenge to sampling methods devoid of detailed sampling frames. In this study, the sampling method was validated in three ways:

First, we compared differences between participants recruited during the first and second sampling phases using Welch’s approximation t-test and Pearson’s Chi-square test for the age and sex distributions, respectively. The median age was 30 years in the first phase and 31 years in the second phase (*p* = 0.93). The gender distribution differed between the two phases, with more women included in the second systematic phase than the first phase (58 and 32%, respectively, *p* = 0.004).

In the second validation analysis, differences between the combined study sample and the camp adult population were calculated. The median age was 30 years in both the combined study sample and the camp population (*p* = 0.11). The combined study sample was 41% women, while the camp population was 46% women (*p* = 0.31).

Finally, selection on the outcome was evaluated in the third validation analysis. The prevalence of MDD did not meaningfully vary between the first and second sampling phases (43% vs. 44%, respectively). We also calculated an age- and sex-standardized MDD prevalence estimate using the population census data. The age- and sex-standardized MDD prevalence estimate was 46%. In this population, consistent with global trends, women had a higher probability of being classified with MDD (54%). The difference between the estimated prevalence of MDD in the combined study sample and the age- and sex-standardized estimate is explained by the slight underrepresentation of women in the study sample.

The differential gender “volunteer” rates in the first phase of sampling are consistent with literature [54]. Systematic sampling in the second phase reached more women, and significantly increased the proportion of women included in the combined sample. We hypothesize that the high response rate of the second phase is in part a reflection of the increased familiarity and positive feedback regarding the research from camp residents and management. In contrast to the frequently transactional nature of humanitarian research, the two-phase sampling approach allowed for increased relational engagement with the study population [47]. An investigation of the effect of the two-phased purposive/systematic approach on the response rate relative to SRS approaches would be a future contribution to research methods for humanitarian emergencies.

## Conclusions

Sampling strategies developed for humanitarian emergencies must maximize protection of the participants, including minimizing coerced consent and the collection of identifiable information, while limiting bias by recruiting and enrolling a representative sample. Combining sampling procedures in humanitarian emergencies can reduce the risk of coerced consent and bias by allowing participants to approach researchers in the first phase, with a second phase of sampling conducted to recruit a systematic sample. This case analysis illuminates the feasibility of a two-phase sampling approach for drawing a quasi-random, representative sample in a refugee camp setting.

## Data Availability

The datasets generated and analyzed during the current study are not publicly available to protect the privacy of the participants, but are available from the corresponding author on reasonable request.

## Abbreviations

95% CI: 95% confidence intervals
AOR: Adjusted odds ratio
EU: European Union
IOM: International Organization for Migration
IQR: Interquartile range
MDD: Major depressive disorder
PHQ-8: 8-item Patient Health Questionnaire
PII: Personally identifiable information
SRS: Simple random sampling
UNHCR: United Nations High Commissioner for Refugees
US: United States
